# The microbiome-restorative potential of ibezapolstat for the treatment of *Clostridioides difficile* infection is predicted through variant PolC-type DNA polymerase III in Lachnospiraceae and Oscillospiraceae

**DOI:** 10.1128/aac.01679-24

**Published:** 2025-02-21

**Authors:** Jacob K. McPherson, Julian G. Hurdle, Matthew L. Baker, Tahir Hussain, Ashok Kumar, Kevin W. Garey

**Affiliations:** 1 Department of Pharmacy Practice and Translational Research, University of Houston 14743 , Houston, Texas, USA; 2 Department of Pharmacological and Pharmaceutical Sciences, University of Houston 14743 , Houston, Texas, USA; 3 Center for Infectious and Inflammatory Diseases, Institute of Biosciences and Technology, Department of Translational Medical Sciences, Texas A&M Health Science Center 26514 , Houston, Texas, USA; 4 Department of Biochemistry and Molecular Biology, McGovern Medical School at the University of Texas Health Science Center 12340 , Houston, Texas, USA; Providence Portland Medical Center, Portland, Oregon, USA

**Keywords:** DNA replication, PolC-type DNA polymerase III, Bacillota, *Clostridioides difficile*, Lachnospiraceae, Oscillospiraceae, ibezapolstat

## Abstract

Ibezapolstat (IBZ), a first-in-class antibiotic targeting the PolC-type DNA polymerase III alpha-subunit (PolC) in low G + C bacteria, is in clinical development for the treatment of *Clostridioides difficile* infection (CDI). In the phase 2 trials, IBZ had potent activity against *C. difficile* while sparing or causing regrowth of Lachnospiraceae, Oscillospiraceae, and Erysipelotrichales, common commensal low G + C bacteria. The purpose of this study was to utilize *in silico* approaches to better interpret the narrower than expected IBZ spectrum of activity. IBZ susceptibility to human commensal microbiota was predicted using genomic analysis and PolC phylogenetic tree construction in relation to *C. difficile* and commensal low G + C bacteria. Protein structure prediction was performed using AlphaFold2 and binding pocket homology modeling was performed using Schrodinger Maestro and UCSF ChimeraX. An amino acid phylogenetic tree identified certain residues that were phylogenetically variant in Lachnospiraceae, Oscillospiraceae, and Erysipelotrichales and conserved in *C. difficile*. Chemical modeling showed that these residues ablated key PolC•IBZ predicted interactions including two lysine “*gates*” (_CdiPolC_Lys1148 and _CdiPolC_Lys1327) that “*latch*” onto the compound; an “*anchoring*” interaction (_CdiPolC_Thr1331) to the central moiety; and a stabilized set of *C. difficile* sensitizer residues (_CdiPolC_Thr1291 and _CdiPolC_Lys1292) that resulted in the prolonged inhibition of a catalytic residue (_CdiPolC_Asp1090). The observed IBZ sparing of Lachnospiraceae, Oscillospiraceae, and Erysipelotrichaceae/Coprobacillaceae was predicted using *in silico* techniques. Further studies that confirm a PolC structural basis for the IBZ narrower than expected activity are needed to confirm these *in silico* phylogenetic and chemical modeling data.

## INTRODUCTION

*Clostridioides difficile* is the most common healthcare-associated pathogen in the United States and causes *C. difficile* infection (CDI) affecting approximately 500,000 patients per year (1). The pathogenesis of CDI involves disruption of a healthy gut microbiome leading to a dysbiotic environment enabling *C. difficile* spores to germinate and cause disease. Antibiotics used to treat CDI can also further disrupt the microbiome contributing to high rates of disease recurrence. Of the two guideline-recommended antibiotics for the treatment of CDI, the RNA polymerase II inhibitor, fidaxomicin (FDX) is a more narrow-spectrum antibiotic on healthy gut commensal organisms than vancomycin (VAN), a glycopeptide antibiotic that inhibits D-ala-D-ala cell-wall synthesis. In head-to-head comparison, FDX was shown to be superior to VAN in the prevention of recurrent CDI (rCDI) (2–4). This supports drug discovery efforts to identify drug targets that kill *C. difficile* without affecting the healthy gut commensal organisms.

Ibezapolstat (IBZ; formerly ACX-362E) is a first-in-class antibiotic that targets the PolC-type DNA polymerase III alpha-subunit (PolC) found in Bacillota and not in other important human gut microbiota phyla including Actinomycetota, Bacteroidota, or Pseudomonadota. IBZ has completed phase 2 clinical trials for the treatment of CDI. Data from the phase 1–2 clinical trials (5–7) showed IBZ minimally disrupted certain Bacillota, specifically Lachnospiraceae, Oscillospiraceae (formerly Ruminococcaceae), and Coprobacillaceae within Erysipelotrichales despite also having the PolC. The reason for this unexpected IBZ sparing of select commensal Bacillota is unknown. We hypothesized that polymorphic differences in PolC among different G + C species would influence IBZ spectrum of activity. *In silico* studies have discovered antibiotic mechanism of action for targeted antibiotics for the fatty acid synthesis protein enoyl-ACP reductase II (FabK) (8). In this regard, we utilized *in silico* methods to better understand this narrower than expected spectrum of activity of IBZ for Lachnospiraceae, Oscillospiraceae, and Erysipelotrichaceae/Coprobacillaceae during therapy.

## MATERIALS AND METHODS

### Protein sequence acquisition and phylogenetic tree construction

Genomic analyses were performed in the CLC Genomics Workbench version 24.0 (Qiagen). A custom microbial database was built comprising 620 RefSeq-deposited reference and representative complete genome assemblies within the phylum Bacillota. From each genome, the *polC* gene was extracted using their automated homology annotations, resulting in 1,113 gene sequences. These 1,113 genes were translated to their respective protein sequences using their coding sequence (CDS) track annotations, resulting in 1,158 protein sequences. These 1,158 protein sequences were further annotated via HMMER (v.3.1b1; May 2013) with Pfam (v.35.0) functional domains to identify the PolC-defining RNaseT (PF00929.27) inserted within the Polymerase and Histidinol Phosphatase (PHP) domain (PF02811.22). Manual screening for protein sequences for this RNaseT insertion within the PHP domain resulted in 620 final PolC sequences (9–11). These 620 PolC sequences were aligned with the CLC Genomics Workbench “Create Alignment” tool (v.1.02) using the very accurate multiple sequence alignment (MSA) algorithm, a gap open cost = 10.0, gap extension cost = 1.0, end gap cost = “as any other,” re-do alignments = “no,” and use fixpoints = “no.” The resulting MSA served as input for the “Create Tree” tool in CLC Genomics Workbench using the Neighbor-Joining algorithm, the Jukes-Cantor distance measure, and 100 replicates of Bootstrapping. The resulting phylogenetic tree was subsequently visualized as a circular phylogram with color-coded to taxonomic family, and node annotations of clinically relevant families. Leaves were manually annotated with general susceptibility to IBZ as either generally IBZ non-susceptible (red), or generally IBZ susceptible (green) based on the results of our metagenomic studies (5, 6).

### Protein structure prediction

In the absence of clinically relevant three-dimensional protein structure data, AlphaFold2 (12) was used to predict the structure of the PolC-type DNA polymerase III (protein ID = CBE03476.1) from the *polC* gene (gene = *dnaF*; locus tag = CDR20291_1146) of the *C. difficile* strain R20291 (NCBI accession = NC_013316.1; GenBank = FN545816.1; RefSeq Assembly = GCF_000027105.1) . Using *C. difficile* strain R20291 PolC (CdiPolC) protein sequence, the three-dimensional structure was predicted via a ColabFold (Google) colab notebook (13). Relevant ColabFold parameters include MSA_method = MMseqs2 (14), pair_mode = “unpaired,” num_relax = 0, use_ptm = “True,” rank_by = “pLDDT” (predicted local distance difference test), num_models = 5, num_samples = 1, num_ensemble = 1, max_recycles = 3, is_training = “False,” and use_templates = “False.” Output quality metrics of prediction accuracy include the MSA coverage, predicted contacts, predicted distograms, predicted alignment error (PAE), the predicted local distance difference test (pLDDT), and a settings log file.

### Molecular docking

The top-ranked AlphaFold2 CdiPolC structure (model 3, pLDDT: 88.5, and pTMscore: 0.7481) served as the input for parallel structure- and template-blind molecular docking of IBZ (PubChem Conformer3D_CID_136022209). The best binding pose was detected using structure- and template-based docking via the CB-Dock2 (15) server that combines CurPocket (16) curvature-based cavity detection with AutoDock-Vina (17, 18) blind docking of the three-dimensional IBZ conformer. The five top-ranked binding poses in CdiPolC cavities were produced, ranked by Vina Score and cavity volume. Visual inspection of the CdiPolC•IBZ complexes was performed in (Schrodinger) Maestro (19, 20) and UCSF ChimeraX (21).

### Binding pocket homology modeling

Using prior knowledge of *Bacillus subtilis azp12* strain resistant to IBZ-predecessor compounds (22), the third rank docked complex was modeled homology modeling of the IBZ binding site near this same active site. The CdiPolC residues that mediated good contacts with IBZ were visually identified using Schrodinger Maestro, and further confirmed by the protein-ligand interaction profiler (23). Following the identification of contact residues, conservation analysis of these residues across two MSAs was performed using CLC Genomics (Qiagen). First, the conservation per residue was analyzed across the same 620 PolC amino acid sequence used above. Second, the conservation of these residues was modeled across 16 representative PolC from 16 clinically relevant species. Data were presented using sequence logos generated via WebLogo (24). Figures were made using BioRender.

## RESULTS

**Fig 1:**
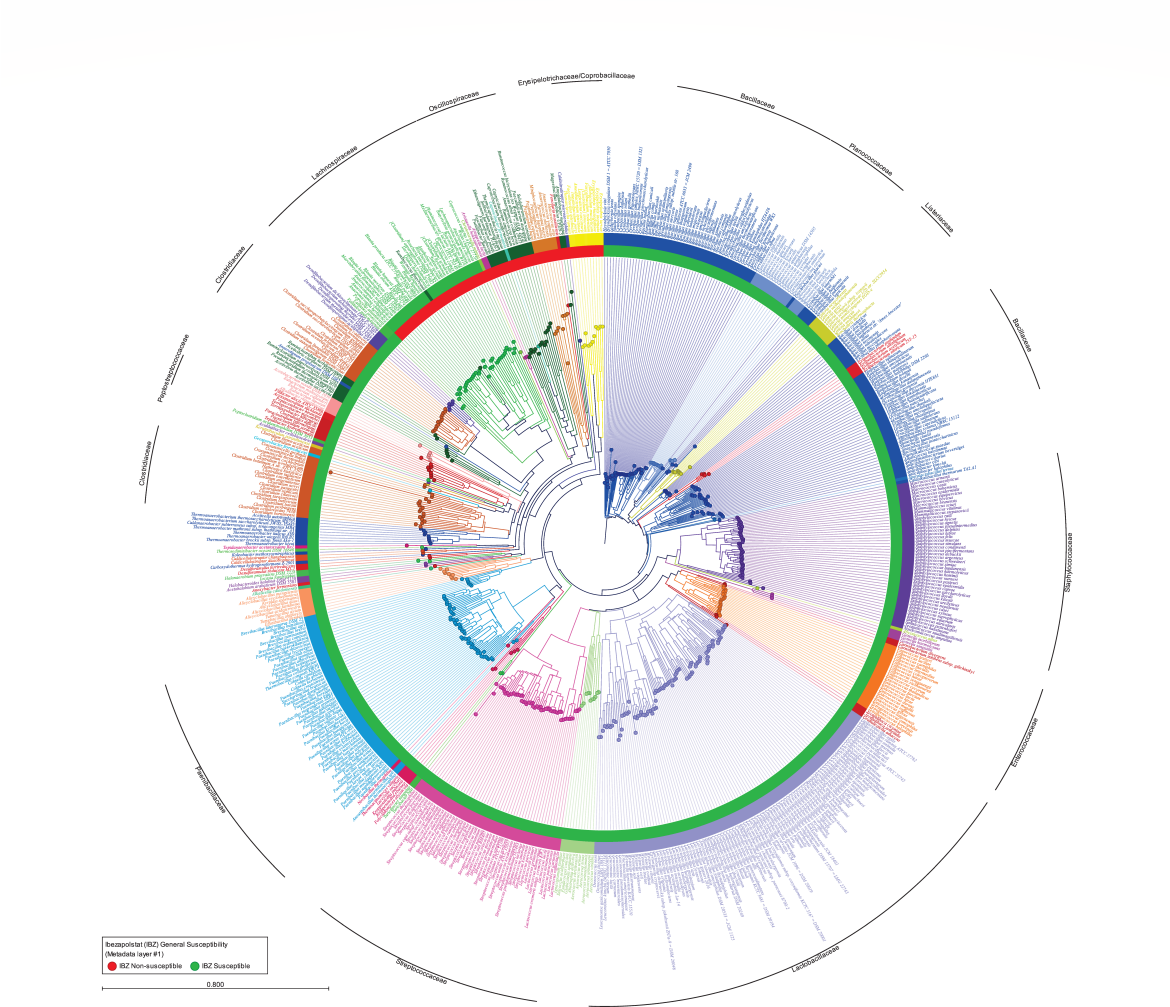
PolC primary sequence relatedness across 620 representative and reference Bacillota. Primary sequence phylogenetic relatedness suggests the PolC from IBZ *non*-susceptible Lachnospiraceae, Oscillospiraceae, and Erysipelotrichales (Erysipelotrichaceae and Coprobacillaceae) are most distal to the phylogenetic root of Bacillaceae PolC.

An amino acid phylogenetic tree was constructed for the PolC from 620 representative Bacillota species (Fig. 1). The tree was annotated with color-coded taxonomic families from the NCBI (colored branches, nodes, outer ring, and name). The intra-phylum phylogenetic relatedness of PolC coincided with the established 16S rRNA evolutionary determinants of taxonomy. Visualization of the tree identified a section of one PolC clade that largely consisted of the Eubacteriales (formerly Clostridiales; coined Clade 3) that contained the IBZ-sparing Lachnospiraceae, Oscillospiraceae, and Erysipelotrichales. Another section of Clade 3 PolC with a root taxonomic family of Thermoactinomycetaceae (cerise magenta) contained Clostridiaceae (*C. butyricum*, *H. histolytica*, *C. septicum*, and *C. sporogenes*) and Peptostreptoccaceae (*C. difficile* and *Paeniclostridium sordellii*) that were generally killed during IBZ-therapy in the phase 2 clinical trial.

### Molecular structure analysis

**Fig 2:**
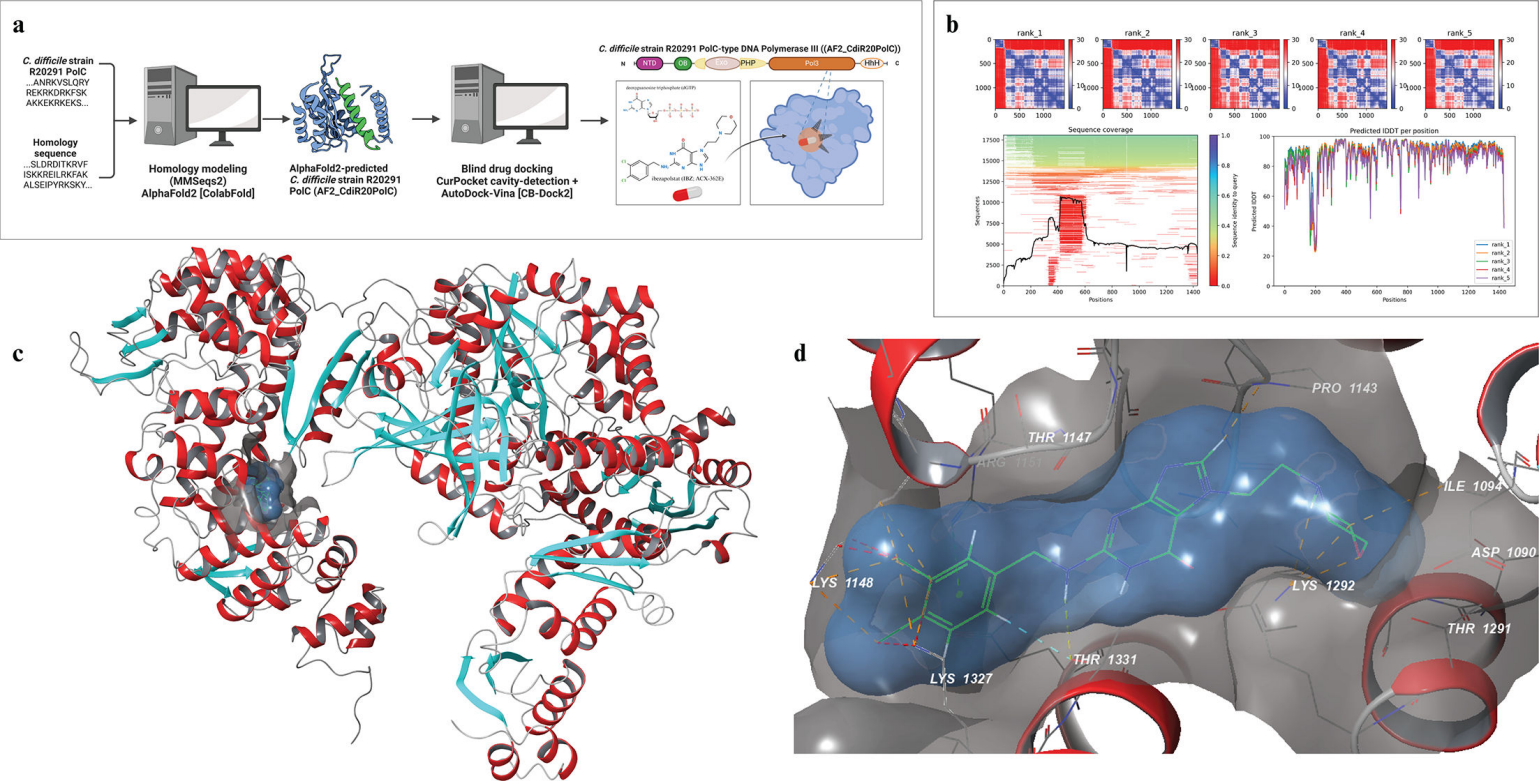
AlphaFold2-based docking with AutoDock-Vina finds the binding pocket and pose of ibezapolstat to the *C. difficile* PolC. (**a**) Overview of AlphaFold2-based docking and the ColabFold notebook with AutoDock-Vina and CB-Dock2 server. (**b**) Predicted aligned error (PAE), sequence coverage, and predicted local distance difference test (pLDDT) per position. (**c**) Macroscopic view of the CdiPolC•IBZ complex. (**d**) Microscopic view of the CdiPolC•IBZ interactions.

To further understand the predicted pharmacological affinity of IBZ, *in silico* template-based cavity detection and structure-based molecular docking via CB-Dock2 were used to identify the binding site pocket and best pose of PolC for *C. difficile*, Lachnospiraceae, Oscillospiraceae, and Erysipelotrichales. A three-dimensional predicted protein structure of the PolC from *C. difficile* strain R20291 (CdiPolC) was generated using a ColabFold notebook running python 3.10 on a Google Cloud A10 GPU using the MMseqs2 sequence alignment algorithm.(Fig. 2) The AF2_CdiPolC quality metric of the pLDDT showed a drop in model confidence around positions 180–200, corresponding to a 20-residue stretch of residues preceding the Exo domain. Otherwise, the majority of the AF2_CdiPolC had a high level of model confidence (average pLDDT 88.5), including the oligonucleotide binding (OB) domain, the duplex binding (DB) domain, and the polymerase palm, thumb, index, and middle fingers.

Given the global confidence of AlphaFold2 in the predicted AF2_CdiPolC, the top-ranked model was docked to IBZ using CB-Dock2 using parallel CurPocket for cavity detection and AutoDock-Vina for virtual docking. Upon visual inspection of the top five docked complexes, one complex whose binding pocket was close to the enzymatic active site of the Polymerase palm where oligonucleotide extension occurs was used for further study. This complex was chosen for further study due to the proximity to the enzymatic active site and prior evidence that the *B. subtilis* azp12 mutant PolC identified a single-amino acid change near this site that confers resistance to the azopyrimidine predecessor compound to IBZ, 6-(p-hydroxyphenylazo)-uracil (HPUra) (22, 25).

### Homology modeling of the binding pocket of PolC

**Fig 3:**
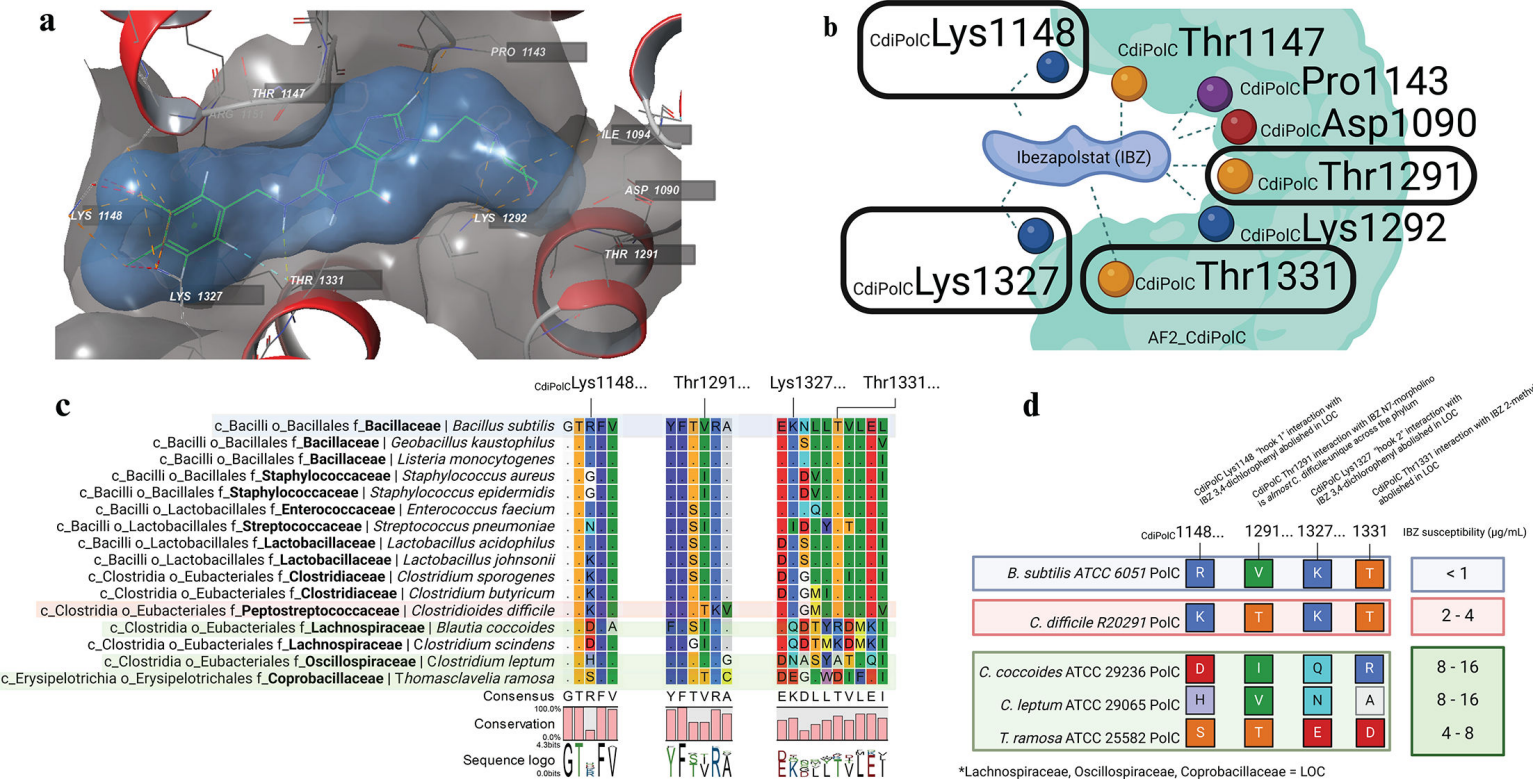
Phylum-wide conservation analysis of the PolC•IBZ binding pocket identifies variant residues and abolished interactions in the Lachnospiraceae, Oscillospiraceae, and Erysipelotrichales PolC: _CdiPolC_Lys1148, _CdiPolC_Thr1291, _CdiPolC_Lys1327, and _CdiPolC_Thr1331. (**a**) Model of key interactions of the AF2_CdiPolC-IBZ complex; variant residues of interest are circled. (**b**) Rotamer selection for tight binding (orange arrows) suggests these four positions latch onto IBZ: sequence logo conservation of 620 representative and reference PolC at these positions. (**c**) Multiple sequence alignment at these four positions across 16 representative Bacillota that correlate with IBZ susceptibility. (d) MIC was determined using agar dilution method.

Visual inspection of the IBZ•AF2_CdiPolC complex using Maestro (Schrodinger) identified PolC residues within 5 Å of IBZ (Fig. 3). Notably, several residues mediated the binding pocket, but only a fraction provided a phylogenetically conserved explanation for the observed IBZ PolC *narrower* spectrum of activity. First, upon visual inspection, two distantly encoded but closely positioned lysine residues, _CdiPolC_Lys1148 and _CdiPolC_Lys1327 were identified near the N2-substituted ((3,4-dichlorophenyl)methyl)amino functional group of IBZ. This would allow electrostatic interaction between the negatively charged chlorines and positively charged nitrogen to lock the two lysine residues in a rare (<10%) rotamer conformation that could bind and hold IBZ. These two lysine “gates” across 16 representative species PolC demonstrated that _CdiPolC_Lys1327 was a highly conserved residue throughout representative PolC species, *except Blautia coccoides* and *C. scindens* (Lachnospiraceae), *C. leptum* (Oscillospiraceae), and *Thomasclavelia ramosa* (Coprobacillaceae). The second lysine “gate,” _CdiPolC_Lys1148, was also a highly conserved residue that followed an evolutionary selection of positively charged arginine or lysine across the Bacillota phylum except *for* the Lachnospiraceae (negatively charged aspartate), the Oscillospiraceae (histidine residue), and the Coprobacillaceae (methionine residue). The _CdiPolC_Thr1327 predicted binding to the polar hydrogens of the IBZ central guanine moiety and via the formation of a hydrogen bond network may have an IBZ “*anchoring*” interaction. This residue is conserved throughout the 16 representative species *except for* the Lachnospiraceae, Oscillospiraceae, and Erysipelotrichales. Finally, the N7-substituted (2-(4-morpholinyl)ethyl) IBZ functional group was in proximity to a handful of potentially interacting residues including _CdiPolC_Thr1291, _CdiPolC_Lys1292, _CdiPolC_Ile1094, and _CdiPolC_Asp1090. Possibly, the morpholino group of IBZ may interact with _CdiPolC_Thr1291 in a *C. difficile*-nearly specific manner that also locks the residue in a rare rotamer conformation. Phylogenetically, _CdiPolC_Thr1291 aligned across the 16 representative species reveals a phylum-conserved preference for aliphatic residues valine or isoleucine, including *B. coccoides* (Lachnospiraceae) and *C. leptum* (Oscillospiraceae). This residue, _CdiPolC_Thr1291, may be a *C. difficile*-specific sensitizer residue specifically or perhaps for other Peptostreptococcaceae. *T. ramosa* also has threonine at this relative position, which may balance its sensitivity to moderately de-sensitized to IBZ, second to Lachnospiraceae and Oscillospiraceae. Finally, aspartates are considered the catalytic residues of DNA synthesis reactions, the proximity of IBZ to _CdiPolC_Asp1090 may explain the competitive inhibition observed in prior studies of steady-state *C. difficile* PolC kinetics (26). Taken together, the homology modeling of the binding pocket of PolC predicted several variant residues that confer IBZ non-susceptibility in Lachnospiraceae, Oscillospiraceae, and Erysipelotrichaceae/Coprobacillaceae.

## DISCUSSION

IBZ is a PolC-type DNA polymerase III alpha-subunit (PolC) inhibitor currently in clinical development for the treatment of CDI. During clinical trials, a narrower than expected spectrum of activity was observed that included increased proportion of certain key microbiota of the Bacillota phylum known to confer health benefits, specifically Lachnospiraceae, Oscillospiraceae (formerly Ruminococcaceae), and Coprobacillaceae within Erysipelotrichales. The purpose of this study was to use *in silico* techniques to hypothesize the mechanism underlying this finding. Recent evidence combining structural biology and phylogenetics of the fidaxomicin-RNAP II interaction identified a single polymorphic “sensitizer residue” at the RNAP β′ (K84) sufficiently confers a more narrow-spectrum of activity to only two of the four phyla of the human gut microbiota (27). We hypothesized that a similar phenomenon could be responsible for the narrow-spectrum activity of IBZ within the Bacillota phylum. The major finding of this study was that the predicted pharmacophore ensemble of interactions between IBZ and PolC (PolC•IBZ) is conserved across the majority of the Bacillota phylum except for Lachnospiraceae and Oscillospiraceae, and Erysipelotrichales (including Erysipelotrichaceae and Coprobacillaceae), taxa that were not killed or regrown in IBZ-treated subjects while on therapy. Within this taxa, residues that were predicted to be phylogenetic variants that may ablate key PolC•IBZ interactions were: two lysine “*gates*” (_CdiPolC_Lys1148 and _CdiPolC_Lys1327) that are predicted to “*latch*” onto the compound; an “*anchoring*” interaction (_CdiPolC_Thr1331) to the central moiety; and a stabilized set of *C. difficile* sensitizer residues (_CdiPolC_Thr1291 and _CdiPolC_Lys1292) that may result in the prolonged inhibition of a catalytic residue (_CdiPolC_Asp1090). While these results will need to be confirmed in experimentally determined structures and molecular genetic approaches, they provide a working hypothesis for the selective narrow spectrum of activity of IBZ.

This study has certain limitations. We used AlphaFold2 with AutoDock-Vina to *in silico* predict key binding site residues IBZ. Despite the accuracy of AlphaFold2 to predict structures from millions of structures from the primary amino acid structures, there are still limitations of the tool. There are only three deposited PolC structures from one species (PDB 3F2B, 3F2C, and 3F2D, *Geobacillus kaustophilus*) for model training. IBZ MICs were higher for commensal bacteria than *C. difficile*; however, these results will need to be confirmed in larger studies using clinical isolates. These results need to be confirmed structurally using the *C. difficile* PolC and in enzymatic, molecular interaction, and cellular genetic assays. Finally, whether the regrowth of these Lachnospiraceae and Oscillospiraceae in IBZ treatment subjects confers a health benefit will require further study.

In conclusion, our *in silico* model predicts that the, *in vivo*, observed IBZ sparing of Lachnospiraceae, Oscillospiraceae, and Erysipelotrichaceae/Coprobacillaceae is due to phylogenetically variant PolC•IBZ binding pocket residues. Further *in vitro* studies that confirm a PolC structural basis for the IBZ narrower than expected activity needed to confirm these *in silico* findings.
